# A Text-Book of the Theory and Practice of Medicine

**Published:** 1893-06

**Authors:** 


A TEXT BOOK OF THE THEORY AND PRACTICE OF MEDICINE, by American Teachers. Edited by William Pepper, M. D., LL.D., Provost and Professor of the Theory and Practice of Medicine and of Clinical Medicine in the University of Pennsylvania. In two volumes, Vol. I. Philadelphia; W. B. Saunders, 913 Walnut street, 1893.

After a careful perusal of the first volume of this new text book, the publishers of the book entitled "American Text Book of Surgery" have issued, we congratulate them that they have come up to the high standard set for themselves. We find here that the teachings of nine colleges are represented, and that these colleges are the most progressive institutions in America. Thirteen authors of international reputation contribute articles on special subjects. The advantage we see in this text book is that it has individuality enough to be the authority of a teacher and yet this individuality is not bounded by any one man.

We live in an age of medical journals, which may be called the text books of the busy practitioner. Here we have the ideas of various minds on various subjects. In the same way, the medical student does not desire a sameness in the treatment of all diseases, and, therefore it is just as well that we have in this form an American Text Book of Practice. In the first volume we find contributions from six writers on forty-five subjects. In the whole work special attention is paid to hygiene, bacteriology, and intestinal parasites, there being charts and illustrations of important morbid processes, etc.

Its character as a text book renders no list of books referred to necessary. But for the fact that it is a large and expensive work, we would expect it to be in general use in medical colleges.

Many a practitioner will be glad to have such a work, if for no other reason, for its attention to preventive medicine to which all treatment should tend, and we think, will reach some day.

On typhoid fever, Dr. Pepper writes one hundred pages and gives place to the following prescription, which should be remembered and kept by every member of the profession.

℞ Ol. terebinthinæ.....................f ʒiij

Pulv. acaciæ.........................

Sacchari.............. aa q. s. ......

Sp. lavandulæ comp..................f ʒiij

Aquæ............ q. s. ad. .........f ℥vi

Ft. mist. sec. artem..................

Sig.—One to two teaspoonfuls in a little water every three hours.

He says in this connection :

Turpentine is, in my judgment, of unquestionable value in certain cases. Not only is it a powerful antiseptic and a good stimulant, but it's action on the mucous membrane in properly selected cases is excellent. J. M. G., JR.

